# LncRNA GAPLINC Promotes Renal Cell Cancer Tumorigenesis by Targeting the miR-135b-5p/CSF1 Axis

**DOI:** 10.3389/fonc.2021.718532

**Published:** 2021-10-14

**Authors:** Siyuan Wang, Xiaorong Yang, Wenjie Xie, Shengqiang Fu, Qiang Chen, Zhilong Li, Zhicheng Zhang, Ting Sun, Binbin Gong, Ming Ma

**Affiliations:** Department of Urology, The First Affiliated Hospital of Nanchang University, Nanchang, China

**Keywords:** long noncoding RNA, GAPLINC, miR-135b-5p, CSF1, renal cell carcinomas

## Abstract

**Background:**

Long noncoding RNAs (lncRNAs) are closely related to the occurrence and development of cancer. Gastric adenocarcinoma-associated, positive CD44 regulator, long intergenic noncoding RNA (GAPLINC) is a recently identified lncRNA that can actively participate in the tumorigenesis of various cancers. Here, we investigated the functional roles and mechanism of GAPLINC in renal cell carcinoma (RCC) development.

**Methods:**

Differentially expressed lncRNAs between RCC tissues and normal kidney tissues were detected by using a microarray technique. RNA sequencing was applied to explore the mRNA expression profile changes after GAPLINC silencing. After gain- and loss-of-function approaches were implemented, the effect of GAPLINC on RCC *in vitro* and *in vivo* was assessed by cell proliferation and migration assays. Moreover, rescue experiments and luciferase reporter assays were used to study the interactions between GAPLINC, miR-135b-5p and CSF1.

**Results:**

GAPLINC was significantly upregulated in RCC tissues and cell lines and was associated with a poor prognosis in RCC patients. Knockdown of GAPLINC repressed RCC growth *in vitro* and *in vivo*, while overexpression of GAPLINC exhibited the opposite effect. Mechanistically, we found that GAPLINC upregulates oncogene CSF1 expression by acting as a sponge of miR-135b-5p.

**Conclusion:**

Taken together, our results suggest that GAPLINC is a novel prognostic marker and molecular therapeutic target for RCC.

## Background

With the expansion of routine imaging examinations of many diseases, an increasing number of patients with renal cell carcinoma (RCC) have been identified (1). Worldwide, RCC ranks sixth in terms of cancer incidence among men and tenth among women, accounting for 5% and 3% of all cancers, respectively (2). Approximately 17% of patients present with metastatic disease at diagnosis (3). Despite the development and wide application of drugs with different mechanisms of action, metastatic renal cell carcinoma (mRCC) is still a highly lethal malignant tumor that presents great challenges (4). Therefore, it is of important clinical value to further explore the possible molecular mechanisms related to the occurrence and development of RCC.

Long noncoding RNAs (lncRNAs) have been identified as RNA molecules with a length of more than 200 bp that lack coding potential (5). In the past few years, the number of tumor-related lncRNAs has increased significantly, and lncRNAs may play a key role at the transcriptional, posttranscriptional or epigenetic level (6). MicroRNAs (miRNAs) are small noncoding regulatory RNAs with sizes of 17-25 nucleotides. After transcription, miRNAs suppress gene expression by identifying complementary target sites in the 3′ untranslated region (UTR) of the target mRNAs (7). MiRNAs are post-transcriptional regulators of gene expression and promising candidates for biomarker development. They play pivotal roles in a wide variety of biological processes, including cell proliferation, survival, differentiation, and tumorigenesis (8). Certain miRNA mimics and miRNA inhibitors have shown promise as novel therapeutic agents (9). Some lncRNAs have a common function; that is, they can act as miRNA decoys to regulate gene expression, which is called the competing endogenous RNA (ceRNA) hypothesis (10). CeRNA hypothesis proposes that the miRNAs can bind to mRNAs, resulting in inactivation for translation, unstable structure and rapid degradation of binded mRNAs. This network links the function of non-coding RNAs to the function of protein-encoding mRNAs (11). The role of ceRNAs in regulating tumor cell proliferation, migration, apoptosis, cell cycle, metastasis, angiogenesis and metabolism has been reported by many laboratories (12).

Gastric adenocarcinoma-associated, positive CD44 regulator, long intergenic noncoding RNA (GAPLINC), a new lncRNA, is abnormally highly expressed in many human tumors and participates in the regulation of malignant biological behavior of tumors (13). MiR-135b-5p can promote or inhibit tumor cell proliferation, migration, invasion and angiogenesis through a variety of biological pathways and can affect the survival and prognosis of tumor patients (14–16). However, the mechanisms of GAPLINC and miR-135b-5p in the occurrence and development of RCC have not been reported. CSF1 is a protein coding gene. The protein it encodes is a cytokine that controls the production, differentiation and function of macrophages (17). Studies have confirmed *in vitro* and *in vivo* that CSF-1 interacts with CSF-1R to promote the survival and proliferation of RCC and reduce apoptosis. Blocking CSF-1R with a CSF-1R tyrosine kinase inhibitor can reduce RCC proliferation and macrophage infiltration, which corresponds to a significant reduction in tumor volume (18–20).

In this study, we found that GAPLINC was highly expressed in RCC tissues and was associated with a poor prognosis. Mechanistic analysis showed that through competitive binding with miR-135b-5p, GAPLINC acts as a ceRNA and regulates the expression of CSF1, thus promoting the proliferation and migration of RCC cells. Overall, our research shows that GAPLINC is a carcinogenic regulatory factor in the occurrence and development of RCC and has potential diagnostic and clinical application value as a therapeutic target.

## Materials and Methods

### RNA Extraction and Transcriptome Data Analysis

Total RNA was extracted from three RCC tissues and three corresponding normal kidney tissues and from three GAPLINC-silenced A498 (renal cancer) cell lines and the corresponding negative control cells using TRIzol reagent (Transgen biotech, China), following the manufacturer’s instructions and then sent to Biotechnology Co., Ltd. (Shanghai, China) and Novogene Co., Ltd. (Beijing, China) for Agilent microarray analysis and sequencing on an Illumina HiSeq 4000 platform. The expression of GAPLINC and CSF1 and patient survival were evaluated in 539 RCC patients and 72 normal controls from the TCGA database (http://tcga-data.nci.nih.gov) using the R programming language.

### Cell Lines and Cell Culture

Human clear cell renal cell carcinoma (ccRCC) cell lines (A498, OSRC-2, ACHN, 786-O and Caki-1) and human renal tubular epithelial cells (HK-2) were purchased from the Chinese Academy of Sciences. OSRC-2 and 786-O cell lines were cultured in RPMI-1640 medium (Gibco, USA), while A498 and ACHN cells were cultured in MEM (Boster, China). The Caki-1 cell line was cultured in McCoy’s 5A medium, and the HK-2 cell line was cultured in DMEM/F12 medium. The medium was supplemented with 10% fetal bovine serum (FBS), penicillin (100 U/mL) and streptomycin (100 µg/mL).

### Real-Time Quantitative PCR

Total RNA was extracted with TRIzol. EasyScript cDNA Synthesis SuperMix (Transgen, China) was used to prepare cDNA. Real-time PCR was conducted in triplicate with TransStart Top Green qPCR SuperMix (+Dye II) (TransGen). **Supplementary Table 1** lists the primers used in this study. β-Actin and U6 were detected as internal controls. The relative amount of RNA was calculated by the 2^−ΔΔCt^ method.

### Subcellular Fractionation

Cytoplasmic and nuclear extracts were prepared in accordance with the manufacturer’s instructions using a cytoplasmic and nuclear RNA purification kit (Norgen, Canada).

### Cell Transfection

SiRNAs and miRNA mimics and inhibitors were synthesized by RiboBio (Guangzhou, China). Cells were transfected with siRNA and miRNA mimics and inhibitors using Lipofectamine 2000 (Invitrogen) following the manufacturer’s protocol. Lentiviruses expressing GAPLINC, sh-GAPLINC and negative control were purchased from HanBio (Shanghai, China). All stably transfected cells were selected with 2 μg/ml puromycin for 48 h.

### Cell Proliferation Assay

The cells were harvested by trypsinization to obtain single-cell suspensions. Then, the cells were quantified by electronic counting (BioRad) and plated in 96-well plates at a density of 3-4 × 10^3^ cells per well. After 24 h, 48 h, 72 h and 96 h of treatment, 10 μl of Cell Counting Kit-8 (CCK-8) solution was added to each well, and the cells were further incubated for 2-4 h. The absorbance values of each sample were measured at 450 nm.

### Wound Healing Assay

When cells in 6-well plates reached confluence, a 200 μl pathogen-free tip was used to scratch the cells. Following washes with PBS, the cells were incubated in medium for 0 h and 24 h at 37°C. Photographs were taken 0 h and 24 h after the scratch, and ImageJ software was used to measure the wound healing capacity.

### Transwell Assay

A total of 3-4×10^4^ cells suspended in 200 µl serum-free medium were seeded into the upper chamber of the Transwell (Corning). Medium containing 15-20% fetal bovine serum (600 µl) was added to the lower chamber. After incubation for 24 h, cells on the lower side were fixed with 4% paraformaldehyde for 15 minutes and stained with crystal violet for 15 minutes. The numbers of stained cells in five random visual fields were counted under a microscope.

### Western Blotting

Proteins were loaded onto 10% SDS-PAGE gels, separated electrophoretically and transferred to PVDF membranes. After blocking in 5% nonfat milk for 1-2 h at room temperature, the membranes were incubated with primary antibody **(** **Supplementary Table 2** **)** overnight at 4°C and subsequently incubated with horseradish peroxidase-conjugated secondary antibody. The Western blotting results were visualized using an enhance chemiluminescence (ECL) detection system and quantified by ImageJ software.

### Luciferase Reporter Assay

Wild-type or mutant 3′-UTR segments of the GAPLINC and CSF1, which are related to the miR-135b-5p binding sites, were cloned downstream of the luciferase gene in pmiR-RB-REPORT™ (RiboBio, China) vector. HEK293T cells of logarithmic growth phase were inoculated in 96-well plates (1.0×10^4^ cells/well), and incubated in incubator for 24 h. Then, cells were cotransfected with GAPLINC-WT/CSF1-WT or GAPLINC-MUT/CSF1-MUT and mimic NC and miR-135b-5p mimic using Lipofectamine 2000 (Invitrogen) according to the manufacturer’s protocols. The transfection medium was replaced 6 h after transfection with fresh complete medium, and cells were collected 48 h later. Luciferase activity for each group was detected using a dual-luciferase reporter assay system (Promega, USA).

### Tumor Xenograft Model

Four-week-old male BALB/c nude mice were purchased from Slake Jingda Laboratory Animal Company (Hunan, China). A498 cells (5 × 10^6^) that had been lentivirally transduced with LV-sh-NC or LV-sh-GAPLINC were suspended in a 100-μL mixture of equal volumes of PBS and Matrigel and injected into the flanks of mice. Tumor volume was measured every 7 days when the tumors were obvious and was calculated using the formula: volume = (length × width^2^)/2 (mm^3^). Studies on animals were conducted with approval from the ethics committee of the First Affiliated Hospital of Nanchang University.

### Immunohistochemistry (IHC)

The tissue samples were fixed in 10% formalin, dehydrated in ethanol, embedded in paraffin and sectioned. The slides were dewaxed, rehydrated, placed in sodium citrate, and microwaved for antigen retrieval. Blocking was performed with 1% BSA, and the sections were then incubated with primary antibodies (anti-Ki-67, anti-MMP-9 or anti-CSF1) at 4°C overnight. Each section was incubated with secondary antibody at 37 °C for 1 h, stained with diaminobenzidine, and counterstained with hematoxylin. Images were captured using a Zeiss microscope and analyzed using ImageJ software.

### Statistical Analyses

Data are presented as the mean ± SD and were analyzed with GraphPad Prism 6 software. Comparisons between groups were performed using unpaired, two-tailed Student’s t tests or the Mann-Whitney U test. Correlations between variables were assessed by the Spearman correlation test. A p < 0.05 indicated statistical significance.

## Results

### GAPLINC Expression Is Upregulated in RCC and Associated With a Poor Prognosis

**Figure 1:**
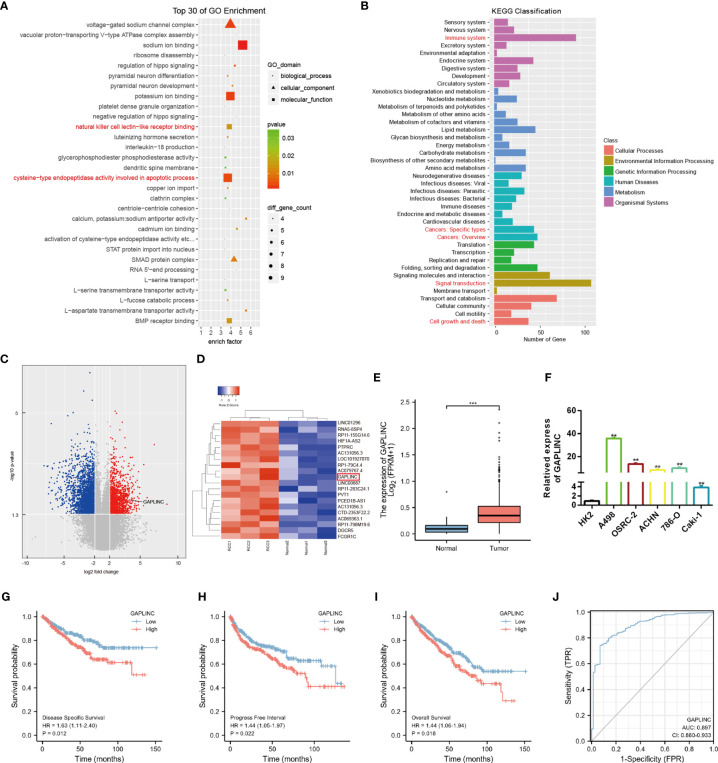
GAPLINC expression is upregulated in RCC and associated with a poor prognosis. **(A)** Top 30 enriched GO terms of the differentially expressed lncRNAs. **(B)** KEGG pathway enrichment analysis of differentially expressed genes. **(C)** The volcano plot shows the expression profiles of lncRNAs. **(D)** Heatmap of the top 20 lncRNAs by expression that are differentially expressed. Red represents high expression, and green represents low expression. **(E)** GAPLINC expression levels in whole tumor tissues and normal tissues in TCGA cohorts. **(F)** RT-qPCR analysis of GAPLINC expression in a human normal renal epithelial cell line (HK-2) and four human RCC cell lines (A498, OSRC-2, ACHN, 786-O, Caki-1). Kaplan-Meier survival analysis according to GAPLINC expression showing the DSS **(G)**, PFI **(H)** and OS **(l)** of RCC patients from the TCGA database. **(J)** ROC curve analysis of the sensitivity and specificity of GAPLINC. **p < 0.01; ***p < 0.001.

To explore the expression profiles of lncRNAs in RCC, high-throughput ceRNA microarray assays of 3 RCC tissues and matched normal kidney tissues were performed. Gene ontology (GO) and Kyoto Encyclopedia of Genes and Genomes (KEGG) analyses demonstrated that the differentially expressed lncRNAs were mainly involved in the immune response and cancer-related biological processes **(** **Figures 1A, B** **)**. The variation in lncRNA expression was revealed in the volcano plot **(** **Figure 1C** **)**. We used the more stringent criteria (p < 0.02, fold change >5) to capture the top 20 upregulated potential candidate genes **(** **Figure 1D** **)**. Then, GAPLINC, which has not been studied in RCC and is highly expressed in RCC tissues and cell lines, was selected as our study object. The lncRNA expression of GAPLINC in 539 RCC tumor tissues and 72 nontumor tissues was analyzed based on the TCGA database **(** **Figure 1E** **)**. RT-qPCR was used to detect the GAPLINC expression levels in the cells, and the results showed that the expression of GAPLINC was higher in tumor cells than in normal cells **(** **Figure 1F** **)**. Kaplan-Meier survival analysis was used to assess the relationship between GAPLINC expression and patient survival (disease-specific survival: DSS, progression-free interval: PFI, and overall survival: OS). Survival analysis showed that high expression of GAPLINC predicted poor DSS, a short PFI and poor OS **(** **Figures 1G–I** **)**. Receiver operating characteristic (ROC) analysis demonstrated that GAPLINC exhibited satisfactory value in RCC diagnosis (AUC = 0.897, 95% CI: 0.860-0.933) **(** **Figure 1J** **)**. Taken together, these results indicate that GAPLINC is upregulated in RCC and associated with a poor prognosis.

### GAPLINC Promotes RCC Cell Proliferation and Migration *In Vitro*

**Figure 2:**
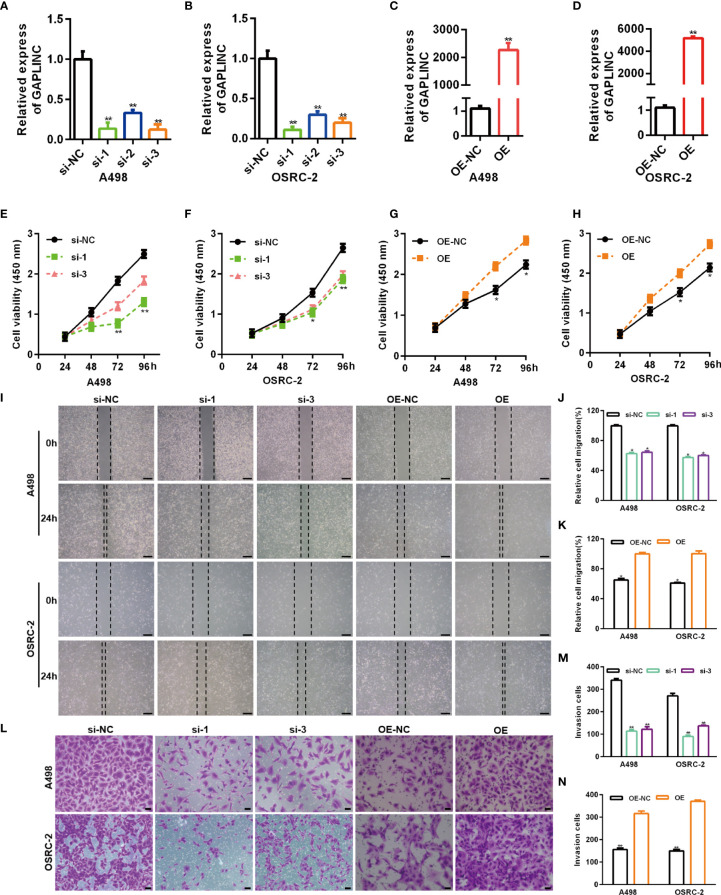
GAPLINC promotes RCC cell proliferation and migration *in vitro*. Validation of the GAPLINC knockdown **(A, B)** and overexpression **(C, D)** efficacy in RCC cell lines by RT-qPCR. **(E–H)** CCK-8 assays in A498 and OSRC-2 cells with silenced or overexpressed GAPLINC. **(I–K)** Wound healing assay of cells after silencing or overexpressing GAPLINC expression. Scale bars, 200 μm. **(L–N)** Transwell migration assays were performed to detect the migration ability when GAPLINC was knocked down or overexpressed. Scale bars, 50 μm. *p < 0.05; **p < 0.01.

RCC cell lines (A498 and OSRC-2) displayed markedly higher GAPLINC expression levels than the kidney normal cell line (HK-2) **(** **Figure 1D** **)**. Thus, these two cell lines were selected for subsequent functional analyses. To further investigate the effect of GAPLINC on cell proliferation, GAPLINC knockdown or overexpression was carried out. We downregulated the expression of GAPLINC in A498 and OSRC-2 cells using small interfering RNA (siRNA). Stable overexpression of GAPLINC was achieved by lentivirus transduction. GAPLINC knockdown and overexpression were confirmed by RT-PCR. As shown in **Figures 2A, B**, the expression levels of GAPLINC were significantly downregulated after transfection with GAPLINC siRNAs (si-1, si-2, and si-3) compared with negative control siRNA (si-NC). In contrast, compared with control vector (OE-NC) transfection, transfection of the GAPLINC overexpression vector (OE) significantly upregulated GAPLINC levels in A498 and OSRC-2 cells **(** **Figures 2C, D** **)**.

We assessed the effects of GAPLINC on cell proliferation using the CCK-8 assay. The results revealed that GAPLINC knockdown remarkably inhibited cell proliferation at 72 h and 96 h compared with that in the si-NC transfection group **(** **Figures 2E, F** **)**. Cell proliferation was increased in the GAPLINC OE group compared to the OE-NC group at 72 h and 96 h (p < 0.05) **(** **Figures 2G, H** **)**. Cell migration was assessed *via* scratch wound healing and Transwell assays. The results indicated that knockdown of GAPLINC inhibited cell migration and that overexpression of GAPLINC promoted the migration of RCC cells **(** **Figures 2I–N** **)**.

### GAPLINC Facilitates RCC Progression by Enhancing CSF1 Expression

**Figure 3:**
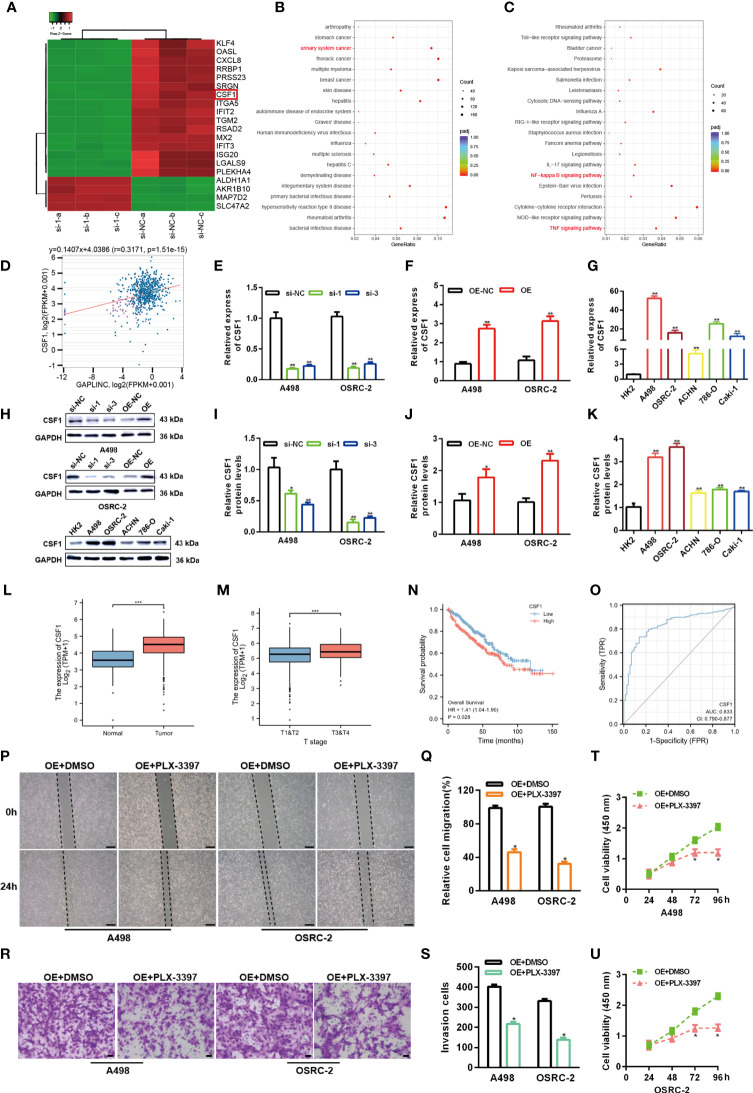
GAPLINC facilitates RCC progression by enhancing CSF1 expression. **(A)** Heat map showing the top 20 transcripts that are altered in A498 cells following GAPLINC silencing. **(B)** DO and **(C)** KEGG enrichment analysis of the differentially expressed genes upon GAPLINC knockdown. **(D)** Pearson correlation analysis between GAPLINC levels and CSF1 levels in RCC tissues according to an online database (http://rna.sysu.edu.cn/chipbase/). CSF1 expression by RT-qPCR after silencing **(E)** or overexpressing **(F)** GAPLINC in A498 and OSRC-2 cells. **(G)** Expression level of CSF1 mRNA in HK-2 and RCC cells measured by RT-qPCR. **(H–K)** Western blotting analysis of the expression of CSF1 protein after silencing or overexpression of GAPLINC and the expression of CSF1 protein in HK-2 and RCC cells. **(L)** TCGA cohort analysis of CSF1 expression levels in RCC samples and adjacent normal tissues. **(M)** CSF1 expression levels in patients with different tumor stages of RCC (TCGA). **(N)** Kaplan-Meier OS curves according to CSF1 expression level. **(O)** The sensitivity and specificity of CSF1 were assessed using ROC curve analysis. Effects of the CSF1 inhibitor PLC-3397 on the migration and proliferation of GAPLINC-overexpressing RCC cells according to wound closure **(P, Q)** (scale bars = 200 μm), Transwell **(R, S)** (scale bars = 50 μm) and CCK-8 assays **(T, U)**. *p < 0.05; **p < 0.01; ***p < 0.001.

To elucidate the potential molecular mechanisms through which GAPLINC contributes to the progression of RCC, we explored the gene expression profiles under GAPLINC silencing conditions. The top 20 genes with the greatest differential expression in GAPLINC-silenced versus control A498 cells were clustered in a heat map plot, and we found that there were many more downregulated genes than upregulated genes **(** **Figure 3A** **)**. DO and KEGG enrichment analyses of downregulated genes was performed to identify the main biological processes **(** **Figures 3B, C** **)**. The results suggested the significant involvement of these genes in the development of RCC. According to ChIPBase v2.0 (http://rna.sysu.edu.cn/chipbase/) coexpression analysis and Pearson’s correlation coefficient analysis, there was a positive correlation between CSF1 expression and GAPLINC expression in 603 RCC samples (r=0.3171, P<0.001) **(** **Figure 3D** **)**. We also confirmed that the expression of CSF1 was higher in RCC cells than in HK-2 cells, and with GAPLINC knockdown and overexpression, CSF1 expression also changed at the transcriptional and protein levels **(** **Figures 3E–K** **)**. Additionally, TCGA database analysis showed that the expression of CSF1 was upregulated in RCC and that CSF1 expression was higher in more advanced RCC tumor stages **(** **Figures 3L, M** **)**. In addition, OS analysis revealed that high CSF1 expression was associated with a poor prognosis, and the area under the curve (AUC) value was suggestive of modest diagnostic value in RCC **(** **Figures 3N, O** **)**. Previous studies showed that CSF1 could act as a promoting regulator in variety of cancers, including RCC (18, 21, 22). Moreover, blocking CSF1 signaling with a CSF1 receptor inhibitor (PLX-3397) has also been shown to suppress tumor progression (23). Our Scratch wound healing experiments and Transwell migration assays indicated that PLX-3397 attenuated the stimulative effects of GAPLINC on RCC cell migration **(** **Figures 3P–S** **)**. CCK-8 assays showed that PLX-3397 attenuated the promoting effect of GAPLINC on RCC cell proliferation **(** **Figures 3T, U** **)**. These results demonstrate that CSF1 is a major candidate target of GAPLINC and that CSF1 plays a major role in mediating the effect of GAPLINC on RCC.

### MiR-135b-5p Inhibits RCC Cell Proliferation and Migration *In Vitro via* CSF1

**Figure 4:**
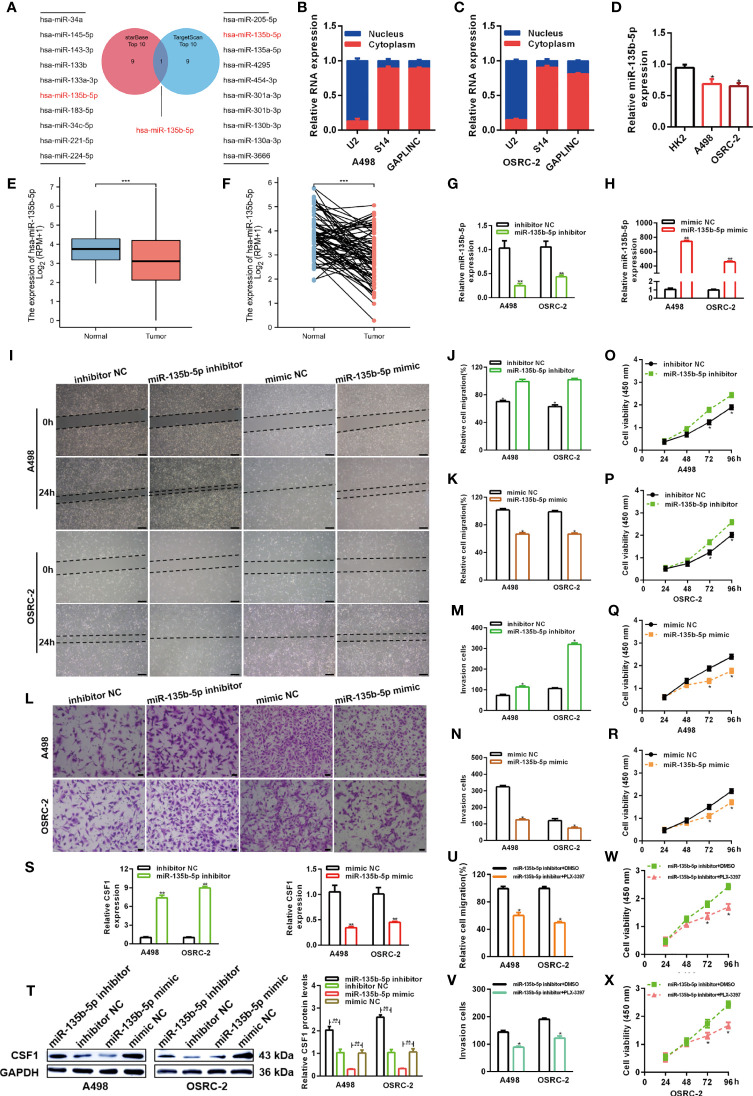
MiR-135b-5p inhibits RCC cell proliferation and migration *in vitro*. **(A)** Schematic showing overlapping miRNAs predicted by the starBase and TargetScan databases to target GAPLINC and CSF1. **(B, C)** Relative level of GAPLINC in the nuclear and cytoplasmic fractions of A498 and OSRC-2 cells. **(D)** Relative expression of miR-135b-5p in A498 and OSRC-2 cells detected using RT-qPCR. TCGA analysis of the expression levels of GAPLINC in paired **(E)** and unpaired **(F)** RCC tissues. MiR-135b-5p expression levels following transfection of A498 and OSRC-2 cells with miRNA inhibitors **(G)** or miRNA mimics **(H)**. The migration capacities were measured by wound closure **(I–K)** (scale bars = 200 μm) and transwell **(L–N)** (scale bars = 50 μm) assays after the cells were transfected with miR-135b-5p inhibitors or mimics. The effect of miR-135b-5p inhibitors **(O, P)** or mimics **(Q, R)** on A498 and OSRC-2 cell proliferation was detected by CCK-8 assay. **(S)** RT-qPCR was performed to detect CSF1 expression after transfection with miR-135b-5p inhibitors and mimics. **(T)** The protein expression level of CSF1 was detected using Western blotting following RCC cell transfection with miR-135b-5p inhibitors or mimics. The effect of PLX-3379 on the migration and proliferation of RCC cells transfected with miR-135b-5p inhibitor was detected by wound closure **(U)**, Transwell **(V)** and CCK-8 **(W, X)** assays. *p < 0.05; **p < 0.01; ***p < 0.001.

The lncRNA-miRNA-mRNA regulatory network, which involves many ceRNAs, has been shown to play a key role in a variety of cancers. Luo et al. predicted the top ten miRNA target sites around the GAPLINC region (24). We also used the TargetScan platform to predict the top ten miRNAs that may target CSF1. Finally, we took the intersection of the results obtained from the two databases and identified miR-135b-5p as a common result **(** **Figure 4A** **)**. Many studies have shown that only lncRNAs located in the cytoplasm can act as molecular sponges to adsorb miRNAs. As shown in **Figures 4B, C**, the subcellular fractionation assays clarified that GAPLINC was mainly located in the cytoplasm. We then detected miR-135b-5p expression in A498 and OSRC-2 cells by RT-qPCR, and the results showed that the expression of miR-135b-5p was downregulated in these cells versus HK-2 cells **(** **Figure 4D** **)**. Moreover, TCGA database analysis showed that miR-135b-5p expression was decreased in non-paired RCC samples **(** **Figure 4E** **)** and paired RCC samples **(** **Figure 4F** **)** compared with normal tissue samples. To explore the function of miR-135b-5p in RCC, we separately transfected A498 and OSRC-2 cells with miR-135b-5p inhibitor or inhibitor NC and miR-135b-5p mimic or mimic NC. The transfection efficiency of the miR-135b-5p inhibitor and mimic was determined by RT-qPCR **(** **Figures 4G, H** **)**. Scratch and Transwell chamber assay results showed that the miR-135b-5p inhibitor promoted the migration of RCC cells, while the miR-135b-5p mimic suppressed their migration **(** **Figures 4I–N** **)**. Additionally, the CCK-8 assay demonstrated that the miR-135b-5p inhibitor increased the proliferation of RCC cells, whereas the miR-135b-5p mimic exhibited the opposite effects **(** **Figures 4O–R** **)**. Moreover, as shown in **Figure 4S**, the miR-135b-5p inhibitor increased the level of CSF1 mRNA, whereas the miR-135b-5p mimic decreased the level of CSF1 mRNA. These results were also verified by Western blotting at the protein level, as shown in **Figure 4T**. Subsequently, scratch wound healing experiments **(** **Figure 4U** **)** and Transwell migration assays **(** **Figure 4V** **)** demonstrated that PLX-3379 attenuated the effect of miR-135b-5p inhibitors on the migration of RCC cells. Similarly, the CCK-8 assay **(** **Figures 4W, X** **)** detected that PLX-3397 could partially reverse the effect of miR-135b-5p on the proliferation of RCC cells. Overall, these results indicate that miR-135b-5p inhibits the migration and proliferation of RCC cells *in vitro via* CSF1.

### GAPLINC Promotes Tumor Progression in RCC *via* the miR-135b-5p/CSF1 Axis

**Figure 5:**
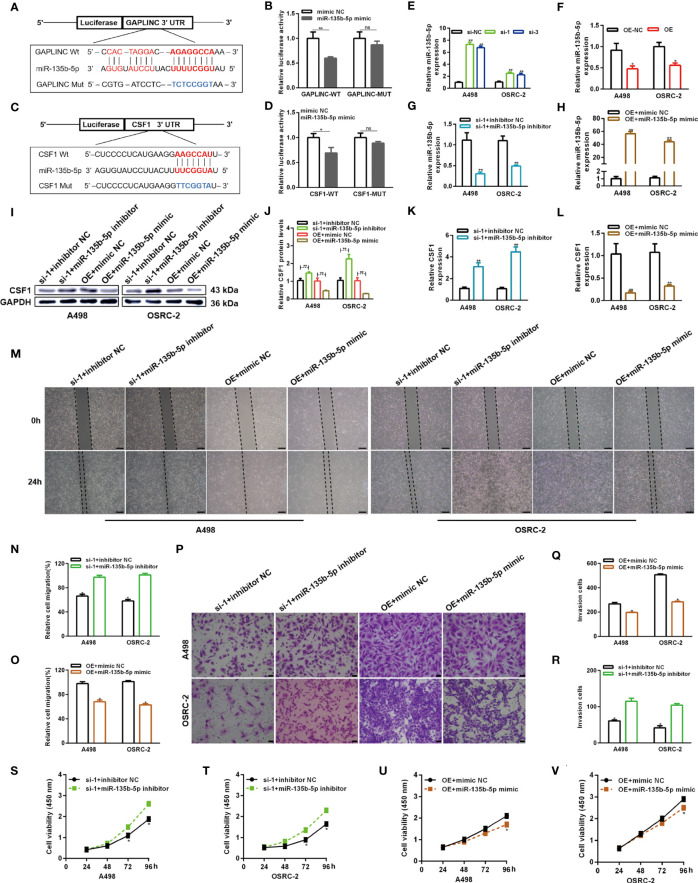
GAPLINC promotes RCC tumor progression *via* the miR-135b-5p/CSF1 axis. **(A–D)** Luciferase reporter assay of A498 cells cotransfected with miR-135b-5p mimics and luciferase reporters containing the wild-type or mutant 3′ UTRs of GAPLINC or CSF1. **(E, F)** MiR-135b-5p expression after silencing or overexpressing GAPLINC in A498 and OSRC-2 cells by RT-qPCR. **(G, H)** The relative expression of miR-135b-5p in RCC cells transfected with si-1/OE vector alone or cotransfected with miR-135b-5p inhibitors/mimics. Western blot **(I, J)** and RT-qPCR **(K, L)** analysis of CSF1 protein and mRNA expression levels after cotransfection with si-1+miR-135b-5p inhibitor or OE vector+135b-5p mimic. Wound healing **(M–O)** (scale bars = 200 μm) and Transwell **(P–R)** (scale bars = 50 μm) assays were employed to investigate the migratory capacity of si-1+miR-135b-5p inhibitor- or OE vector+135b-5p mimic-cotransfected RCC cells. The proliferation ability of RCC cells cotransfected with si-1+miR-135b-5p inhibitor **(S, T)** or OE vector+135b-5p mimic **(U, V)** was determined by CCK-8 assays. *p < 0.05; **p < 0.01; ns, no significance.

Numerous studies have shown that cytoplasmic lncRNAs can act as effective miRNA sponges, as they contain conserved miRNA target sites, competitively inhibiting miRNAs to regulate downstream target gene expression at the posttranscriptional level. The online miRNA databases RNAhybrid and TargetScan were used to reveal potential binding sites between miR-135b-5p and GAPLINC and between miR-135b-5p and CSF1, respectively. Then, we performed a dual-luciferase reporter assay to verify their paired binding. Then, to verify this, the sequences of GAPLINC-Wt, GAPLINC-Mut, CSF1-Wt or CSF1-Mut were inserted into the luciferase reporter plasmid **(** **Figures 5A, C** **)**. Then, Wt and Mut reporter constructs were cotransfected into HEK 293 cells with miR-135b-5p mimic or mimic NC as indicated. The results showed that only the miR-135b-5p mimic effectively inhibited the luciferase activity of the Wt group compared with the other groups **(** **Figures 5B, D** **)**. In addition, RT-qPCR experiments revealed that knockdown of GAPLINC promoted the expression of miR-135b-5p, whereas GAPLINC overexpression decreased miR-135b-5p expression **(** **Figures 5E, F** **)**. These results show that GAPLINC can bind to miR-135b-5p and that there is a direct relationship between miR-135b-5p and CSF1. Next, we performed miR-135b-5p inhibitor or inhibitor NC and GAPLINC si-1 cotransfection and miR-135b-5p mimic or mimic NC cotransfection with the GAPLINC OE vector, to study whether the effect of GAPLINC on CSF1 expression is dependent on miR-135b-5p. Cotransfection of miR-135b-5p inhibitor and si-1 led to a decrease in miR-135b-5p expression compared with that in the inhibitor NC + si-1 group. In addition, the expression of miR-135b-3p was increased in the OE vector + miR-135b-5p mimic group compared with the OE + mimic NC group **(** **Figures 5G, H** **)**. We quantitatively detected the effect of cotransfection on the expression of CSF1 at the protein expression and transcriptional levels. The results showed that cotransfection with the miR-135b-5p inhibitor reversed the downregulation of CSF1 mRNA and protein expression mediated by si-1. However, cotransfection with the miR-135b-5p mimic reversed the upregulation of CSF1 mRNA and protein expression mediated by the OE vector **(** **Figures 5I–L** **)**. To further explore the functional significance of the miR-135b-5p/CSF1 axis in the protumor role of GAPLINC in RCC, we conducted rescue experiments. We observed that the miR-135b-5p inhibitor attenuated the deceases in the migration and proliferation of cells induced by GAPLINC knockdown. In contrast, the miR-135b-5p mimic weakened the promoting effect of GAPLINC overexpression on the migration and proliferation of A498 and OSRC-2 cells **(** **Figures 5M–V** **)**. All these data demonstrate that GAPLINC promotes the proliferation and migration of RCC cells *in vitro*, partly by sponging miR-135b-5p, thereby regulating CSF1 levels.

### Knockdown of GAPLINC Inhibits the Tumorigenicity of RCC *In Vivo*

**Figure 6:**
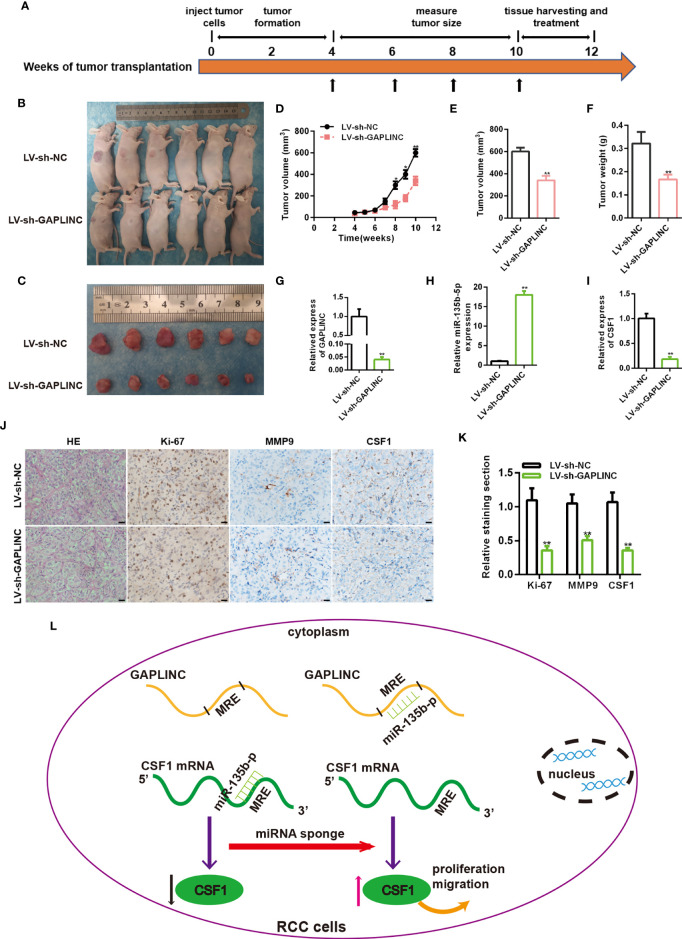
Knockdown of GAPLINC inhibits the tumorigenicity of RCC *in vivo*. **(A)** Schematic representation of the nude mouse xenograft tumor experimental protocol. **(B, C)** Photographs of tumors obtained from the LV-sh-NC and LV-sh-GAPLINC groups of nude mice. The tumor volumes were measured once a week after tumor formation **(D)**, and the tumor nodules were removed 10 weeks after transplantation, after which the final volumes **(E)** and weights **(F)** were measured. Xenograft tissues were subjected to RT-qPCR to assess GAPLINC **(G)**, miR-135b-5p **(H)** and CSF1 **(I)** expression. **(J, K)** Representative images showing H&E, Ki-67, MMP-9 and CSF1 staining of xenograft tumor tissues; the histogram shows the statistical analysis of their expression levels. Scale bars, 20 μm. **(L)** Schematic representation of the mechanism of GAPLINC in RCC cells. *p < 0.05; **p < 0.01.

To further evaluate the functional role of GAPLINC in the growth of RCC tumors *in vivo*, A498 cells stably transduced with LV-sh-NC or LV-sh-GAPLINC were subcutaneously inoculated into nude mice. The scheme of the experimental procedure is outlined in **Figure 6A**. As shown in **Figures 6B, C**, we examined orthotopic tumors under the skin and tumors resected from nude mice. According to the analysis of tumor volume over time, we observed that from the second week, the tumor volume was effectively lower in the LV-sh-GAPLINC group than in the LV-sh-NC group **(** **Figure 6D** **)**. At 10 weeks after tumor implantation, GAPLINC knockdown had dramatically reduced the tumor volume and the tumor weight compared with that in the control group **(** **Figures 6E, F** **)**. Then, RT-qPCR was performed to measure the expression levels of GAPLINC, miR-135b-5p and CSF1 in tumor tissue specimens from engrafted nude mice. The results showed that GAPLINC and CSF1 mRNA levels in the LV-sh-GAPLINC group were lower than those in the LV-sh-NC group **(** **Figures 6G, I** **)**. However, the miRNA level displayed the opposite trend (**Figure 6H**). Hematoxylin and eosin (H&E) staining and immunohistochemistry staining analysis of CSF1, Ki-67 and MMP-9 expression in tumors from xenograft mice of the two groups. The results confirmed that the expression of Ki-67, MMP-9 and CSF1 was decreased in tumors upon GAPLINC downregulation **(** **Figures 6J, K** **)**. The above results indicate that GAPLINC can also promote the proliferation and migration of RCC *in vivo*, which is consistent with our *in vitro* results.

## Discussion

Renal cell tumors are a group of histopathologically and molecularly heterogeneous tumors with different genetic and epigenetic abnormalities (25, 26). With the development of sequencing technology, it is possible to detect hundreds of molecular biomarkers in clinical samples. Therefore, further research on molecular biomarkers in RCC is needed to facilitate diagnose and prognostic risk stratification and to identify potential treatment targets for patients with advanced disease (27). The study of the molecular pathogenesis of RCC is used to determine the target of treatment intervention to guide the choice of treatment for each patient in a personalized approach. This has led to the development of a variety of drugs that play an important role in the management of mRCC. Drugs targeting the vascular endothelial growth factor (VEGF) pathway and drugs inhibiting the mammalian target of rapamycin (mTOR) protein have been used in the treatment of mRCC. In addition, the recent emergence of immune checkpoint inhibitors has led to significant changes in mRCC treatment. The PD-1 inhibitor nivolumab has been shown to improve the OS of mRCC patients after VEGF inhibitor treatment (28). As these types of sequencing-based assays are incorporated into routine clinical care, they have the potential to fundamentally change the way we manage renal cell carcinoma (29).

From the perspective of genome and transcriptome expansion, our catalog of genetic elements is now full of lncRNAs. It is estimated that the number of human lncRNAs exceeds the number of protein-coding genes (30). The functions of most lncRNAs are not clear, and many lncRNAs may not have obvious functions, but the functions and mechanisms of some classically defined lncRNAs, such as XIST and HOTAIR, are already clear (31). LncRNAs have been discovered to function as scaffolds, decoys or signals that can function through genomic targeting, cis or trans regulation and antisense interference (32). Like proteins, lncRNAs must be located in a specific subcellular compartment to perform their functions. For example, the lncRNA NKILA, a cytoplasmic lncRNA, can interfere with protein posttranslational modification, resulting in abnormal signal transduction (33). In addition, cytoplasmic lncRNAs such as linc-MD1 (34) and lncRNA NORAD (35) can affect gene regulation by acting as decoys for miRNAs and proteins. Quantitative analysis of the abundance of miRNAs and target mRNAs shows that the ceRNA mechanism of cytoplasmic lncRNAs can work only when there is proper stoichiometry between lncRNAs and miRNAs. Therefore, in addition to matching miRNA seed sequences, experimental analysis and knockout studies are needed to verify the ceRNA mechanism (36).

Evidence suggests that lncRNAs may be involved in almost all human cancers and may play a role in stem cell maintenance, cell proliferation, apoptosis, cell invasion and metastasis (37, 38). The latest progress in the study of the molecular mechanism of lncRNAs provides a tool for the functional annotation of these cancer-related transcripts, making these molecules an attractive target for cancer therapy and intervention (39). As many as 35000 different lncRNAs have been found in RCC. However, only hundreds to thousands of these lncRNAs exhibit differential expressions in RCC tissues and normal renal tissues (40). Malouf et al. identified 1934 abnormally expressed lncRNAs in RCC and established the first genome-wide classification of RCC-related lncRNAs, revealing their correlation with clinicopathology and genomic characteristics (41). Because lncRNAs have tissue-specific expression compared with mRNAs, the interaction between lncRNAs and their binding proteins or miRNAs can be blocked by antisense oligodeoxynucleotides and small molecular compounds, and abnormally expressed lncRNAs can be targeted to treat RCC (42).

Hu et al. reported in 2014 that a new lncRNA located on human chromosome 18p11.31 was found in gastric cancer and affects the proliferation and migration of gastric cancer cells by sponging miR-211-3p to regulate the expression of CD44; this lncRNA was named GAPLINC (43). Subsequently, GAPLINC was found to be highly expressed in hepatocellular carcinoma (44) and non-small-cell lung cancer (45) and was found to promote cancer through different mechanisms. Vollmers et al. found that GAPLINC knockout mice showed resistance to endotoxic shock induced by lipopolysaccharide (46). At the same time, Mo and other studies have shown that GAPLINC can promote the tumor-like biological behavior of fibroblast-like synoviocytes in patients with rheumatoid arthritis through a ceRNA mechanism (47). As a member of the miRNA family, miR-135b-5p plays a role in a variety of cancers. Studies have shown that miR-135b-5p is upregulated or downregulated in cancer tissues and plays a role in promoting or inhibiting cancer through a variety of mechanisms (14, 48–50). Similarly, miR-135b-5p can also lead to neuronal damage after stroke (51) and contributes to rheumatoid arthritis (52) by participating in the inflammatory response. The present study reported for the first time that GAPLINC was upregulated in RCC tissues and was associated with a poor prognosis. The relationship between the expression levels of GAPLINC and miR-135b-5p was proven by RT-qPCR. GAPLINC silencing led to increased miR-135b-5p expression in RCC cells. However, overexpression of GAPLINC gave the opposite result. Cell experiments *in vitro* confirmed that GAPLINC promotes the proliferation and migration of RCC cells, while miR-135b-5p does the opposite. Animal experiments also show that GAPLINC promotes the proliferation of RCC and makes Ki-67 highly expressed. We further discovered that the protein levels of MMP9 were decreased after GAPLINC knockdown in the tumor tissues of nude mice. It indicates that GAPLINC promotes cell migration in animal models, which may be related to the high expression of MMP9, which plays an important role in extracellular matrix remodeling and membrane protein cleavage.

The tumor microenvironment (TME) is made up of noncancer cells in the tumor, including fibroblasts, immune cells, cells that make up blood vessels and proteins produced by cells that support the growth of cancer cells (53). Cancers develop in complex tissue environments in which they can continue to grow, invade and metastasize. Unlike tumor cells, the stromal cells in the TME are genetically stable, so they provide attractive therapeutic targets for reducing drug resistance and the risk of tumor recurrence (54). Macrophages are the most abundant cells in the tumor stroma and have obvious plasticity, so they can play a variety of roles in the TME. Tumor-associated macrophages (TAMs) usually refer to M2 macrophages, which have anti-inflammatory and protumor effects. M2 macrophages can also neutralize inflammation-promoting effects and the antitumor M1 phenotype. The shift of macrophages to the anti-inflammatory M1 phenotype can be used as an adjuvant approach with other methods including radiotherapy and immune checkpoint blockade, such as anti-PD-L1/PD-1 strategies (55).

Evidence suggests that noncoding RNAs (ncRNAs) can guide the development of a variety of immune cells and control dynamic transcriptional procedures (56). Linear ncRNAs, such as lncRNAs and miRNAs, have been found to play an important role in the regulation of tumor immunity and immunotherapy (57). As the core of a wide range of regulatory elements that control the inflammatory response circuit, lincRNA-Cox2 mediates the activation and inhibition of different kinds of immune genes (58). Li et al. showed that miR-146a can promote the growth of subcutaneous breast tumors in mice by promoting M2 polarization or regulating the recruitment of TAMs (59). RCC is an effective immunotherapy disease, and it is worthwhile to study whether lncRNAs can regulate the immune pathway of RCC (60). Because linear ncRNA has its own function in regulating the response to different immunotherapies, the use of ncRNA as an adjuvant for immunotherapy has great potential (61).

Macrophage colony stimulating factor (CSF-1) is a hematopoietic growth factor that is related to the survival, proliferation and differentiation of macrophages, monocytes and bone marrow progenitor cells. After CSF-1 treatment, macrophages polarize to the M2 phenotype (62). Pyonteck et al. used CSF-1R inhibitors to target TAMs in mouse glioma models. The results showed that the inhibitor slowed down the intracranial growth of patient-derived glioma xenografts and significantly increased the survival time (63). CSF-1 has been shown to interact with CSF-1R to promote the survival and proliferation of RCC cells and reduce apoptosis. *In vivo*, the use of a CSF-1R tyrosine kinase inhibitor to block CSF-1R reduced RCC proliferation and macrophage infiltration, which was related to a significant reduction in tumor volume (18). PLX-3397 (pexidartinib) is an oral, effective, CSF-1R inhibitor that has entered the stage of clinical development. The clinical efficacy of it as a single treatment or adjuvant therapy is being evaluated (23). In this study, we observed that CSF1 is a target protein of the GAPLINC/miR-135b-5p axis. Through dual-luciferase reporter gene analysis, it was proven that CSF1 is a direct target gene of miR-135b-5p. In addition, we showed that overexpression of GAPLINC led to increased expression of CSF1, which was partially reversed by the overexpression of miR-135b-5p.

In brief, we identified the high expression of GAPLINC in RCC and confirmed that it was associated with a poor clinical prognosis. *In vitro* proliferation, scratch, Transwell assays and *in vivo* experiments proved that GAPLINC promoted the proliferation and migration of RCC cells. Low expression of CSF1 protein was found after GAPLINC knockdown. Phenotypic experiments proved that GAPLINC plays a role in promoting cancer through CSF1. Subsequently, the ceRNA mechanism by which GAPLINC upregulates CSF1 by sponging miR-135b-5p was identified by luciferase assays and phenotypic rescue experiments. Overall, we elucidated the significance of the GAPLINC/miR-135b-5p axis in RCC **(** **Figure 6L** **)**. This may provide a new perspective for the molecular targeted treatment of RCC.

## Conclusion

Our study revealed that the high expression of GAPLINC in RCC is associated with a poor prognosis. GAPLINC increases the expression of CSF1 by sponging miR-135b-5p, thus promoting the proliferation and migration of RCC cells. Our study further deepens the understanding of the pathogenic roles of lncRNAs in RCC and provides a potential new therapeutic target for patients with RCC.

## Data Availability Statement

The original contributions presented in the study are included in the article/**Supplementary Material**. Further inquiries can be directed to the corresponding authors.

## Ethics Statement

The studies involving human participants were reviewed and approved by Institutional Review Board of the First Affiliated Hospital of Nanchang University. The patients/participants provided their written informed consent to participate in this study. The animal study was reviewed and approved by Animal Care and Use Committee of the First Affiliated Hospital of Nanchang University, China.

## Author Contributions

TS, BG, and MM designed the experiments. SW performed the experiments. XY and WX analyzed the data. SF, QC, ZL, and ZZ interpreted the results. SW, BG, and MM wrote the paper. All authors contributed to the article and approved the submitted version.

## Funding

This work was supported by the National Natural Science Foundation of China (91200212), the Provincial Natural Science Foundation of Jiangxi (20202BABL206023), China and Graduate Student Innovation Special Fund Project of Jiangxi Province (YC2020-B042).

## Conflict of Interest

The authors declare that the research was conducted in the absence of any commercial or financial relationships that could be construed as a potential conflict of interest.

## Publisher’s Note

All claims expressed in this article are solely those of the authors and do not necessarily represent those of their affiliated organizations, or those of the publisher, the editors and the reviewers. Any product that may be evaluated in this article, or claim that may be made by its manufacturer, is not guaranteed or endorsed by the publisher.
