# Supplementary material for: LncRNA GAPLINC Promotes Renal Cell Cancer Tumorigenesis by Targeting the miR-135b-5p/CSF1 Axis
Source: Front Oncol. 2021 Oct 14;11:718532. doi: 10.3389/fonc.2021.718532 (PMC8551964; doi:10.3389/fonc.2021.718532)
Supplement: Supplementary file 1 [file Table_1.docx]

Supplementary Table 1. The primers for RT-qPCR

| **Gene** | **Forward or Reverse** | **Primer sequence** |
| --- | --- | --- |
| GAPLINC | Forward | 5’-TGGACTCAGGCACGTTTACAG-3’ |
|  | Reverse | 5’-TCATTGTTCTGGCCTCTGTCC-3’ |
| CSF1 | Forward | 5’-CAGGAGGAGCCCCAGAG-3’ |
|  | Reverse | 5’-CAGTGCGTGAGCCAATGT-3’ |
| U2 | Forward | 5’-CATCGCTTCTCGGCCTTTTG-3’ |
|  | Reverse | 5’-TGGAGGTACTGCAATACCAGG-3’ |
| S14 | Forward | 5’-GGCAGACCGAGATGAATCCTC-3’ |
|  | Reverse | 5’-CAGGTCCAGGGGTCTTGGTCC-3’ |

Supplementary Table 2. Antibodies used for WB or IHC

| **Antibody** | **Description** | **Supplier** | **City** | **Country** |
| --- | --- | --- | --- | --- |
| Anti-CSF1 | Rabbit | AbCam(ab233387) | Cambridge | USA |
| Anti-ki67 | Rabbit | servicebio(GB111141) | Wuhan | China |
| Anti-MMP9 | Rabbit | CST(13667S) | Boston | USA |
